# Feasibility and acceptability of pediatric expert teleconsultation during critical ambulance transports: a pilot randomized controlled simulation trial

**DOI:** 10.1016/j.resplu.2026.101448

**Published:** 2026-08-14

**Authors:** Tehnaz P. Boyle, Derrick Chu, Ijeoma M. Okafor, Lauren Cronin, Caitlin Farrell, Stephanie Stapleton, Ron Medzon, Barbara M. Walsh, Howard Cabral, Erik Wang, Mari-Lynn Drainoni, Vinay Nadkarni, Carlos A. Camargo, James A. Feldman

**Affiliations:** a Department of Pediatrics, Boston Medical Center, Boston University Chobanian & Avedisian School of Medicine, Boston, USA; b Department of Pediatrics, Boston Children’s Hospital, Harvard Medical School, Boston, USA; c Department of Emergency Medicine, Boston Medical Center, Boston University Chobanian & Avedisian School of Medicine, Boston, USA; d Department of Biostatistics, Boston University School of Public Health, Boston, USA; e Department of Emergency Medicine, Lahey Hospital & Medical Center, Tufts University School of Medicine, Boston, USA; f Section of Infectious Diseases, Department of Medicine, Boston Medical Center, Boston University Chobanian & Avedisian School of Medicine, Boston, USA; g Department of Health Law Policy & Management, Boston University School of Public Health, Boston, USA; h Evans Center for Implementation and Improvement Sciences, Department of Medicine, Boston University Chobanian & Avedisian School of Medicine, Boston, USA; i Department of Anesthesiology, Critical Care, and Pediatrics, Children’s Hospital of Philadelphia, University of Pennsylvania Perelman School of Medicine, Philadelphia, USA; j Department of Emergency Medicine, Massachusetts General Hospital, Harvard Medical School, Boston, USA

**Keywords:** Teleconsultation, Pediatric, Emergency Medical Services, Ambulance

## Abstract

**Background:**

Before a trial testing prehospital teleconsultation for children, we studied acceptability, feasibility, and workload after integrating teleconsultation into Emergency Medical Service (EMS) workflows.

**Methods:**

We randomized paramedic-physician teams to teleconsultation (audio/video call, intervention) or usual care (audio call, control) for three simulated pediatric transports using mixed methods. Paramedic dyads provided in-ambulance simulated care while remote pediatric emergency physicians offered real-time consultation. In the intervention group, we used the Telehealth Usability Questionnaire to measure acceptability [primary; Total Usability Score (TUS), maximum = 7] and feasibility [secondary; Interaction Quality Score (IQS)]; and interviews for Technology Acceptance Model themes. Between groups, we calculated descriptive statistics and compared connection success and workload.

**Results:**

Twenty-four teams (12/group) completed 72 simulated transports. Intervention paramedics attempted physician contact in 94% (34/36) of transports versus 47% (17/36) in control; 92% connected in ≤2 attempts. In the intervention group, mean TUS was 5.6 (95% CI 5.2, 6.0) for physicians and 5.8 (95% CI 5.5, 6.0) for paramedics; mean IQS was 5.2 (95% CI 4.6, 6.0) and 5.9 (95% CI 5.4, 6.3) respectively. Intervention paramedics reported similar workload to controls (*p* = 0.26) but less frustration (*p* = 0.03); physicians experienced higher workload (*p* = 0.01). Interviews identified improved communication and physician involvement, alongside uncertainty about physician roles and impact on paramedic autonomy. Video quality was adequate, but concerns about real-world audio and broadband reliability remained.

**Conclusions:**

In simulations, teleconsultation for pediatric EMS transports was acceptable and feasible with minor technical modifications. Implementation should balance physician workload and clarify roles through training. Future trials should evaluate patient outcomes.

## Introduction

Emergency Medical Service (EMS) readiness is necessary for safe and effective care of children.^1^ Of the 3 million children transported annually in the United States (U.S.), 10–14% requires lifesaving care outside hospitals.^2^ Prehospital care quality and outcomes are worse for critically ill children than adults, partly because these high-stress encounters are rare.^3^, ^4^, ^5^, ^6^, ^7^ Interventions to support EMS clinicians could reduce preventable harm and improve outcomes.^8^, ^9^

The EMS for Children Program and other national initiatives have prioritized prehospital care safety and quality.^1^, ^10^ Whether national efforts to expand equipment, protocols, and training will increase EMS provider preparedness is unclear.^1^, ^11^, ^12^ Durability may be limited by the infrequency of pediatric critical illness compared to adults, consolidation of pediatric specialists in tertiary centers, and rising medical complexity of children.^4^

Real-time clinician support with pediatric experts offers a complementary solution. Pediatric teleconsultation could address gaps in EMS pediatric readiness by reducing risk of errors and supporting decision-making in time-sensitive emergencies. However, most U.S. EMS systems use audio-based communication (radio or telephone) for physician oversight.^13^ Teleconsultation allows physicians to view patients remotely and assist with assessment, diagnosis, or treatment in real-time.^14^ EMS telehealth programs have improved select adult outcomes,^15^, ^16^, ^17^ but adoption has lagged without efficacy data^18^, ^19^ amid concerns about cost, broadband availability, and transport delays.^20^, ^21^ Whether benefits of hospital-based teleconsultation for children^22^, ^23^, ^24^, ^25^ extend to prehospital care is unknown.

To inform a future efficacy trial, we assessed the acceptability of using off-the-shelf mobile technology for teleconsultation during simulated pediatric transports, and compared the feasibility of establishing video connection and perceived clinician workload to usual audio-based methods of physician consultation (usual care). Because such interventions can alter clinician behavior and disrupt critical care, we used in situ simulation in real care environments to test teleconsultation during simulated pediatric transports to minimize patient and clinician risk.

## Methods

### Study design

**Fig. 1:**
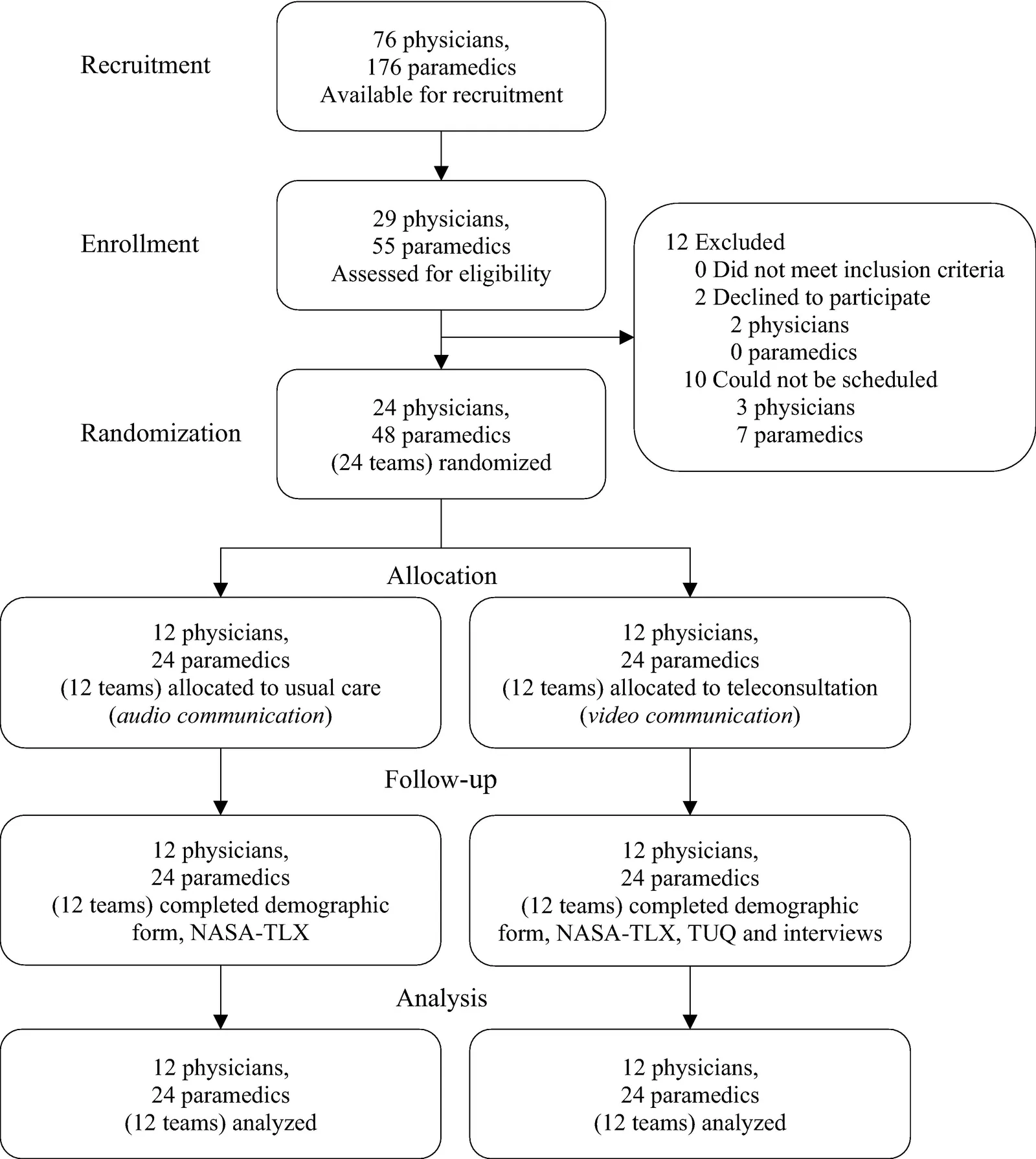
**Study flow diagram**.

This single-blind, parallel-arm, pilot simulation randomized controlled trial used mixed methods (Fig. 1). We measured (1) feasibility and workload with observation and surveys of both groups, and (2) acceptability with questionnaires and semi-structured interviews of the intervention group. The Boston Medical Center Institutional Review Board approved this study (H-37817). This report uses CONSORT guideline extensions for simulation research^26^ and randomized pilot trials.^27^

### Study sites

We conducted simulations at five EMS agencies in the Boston area (Supplemental Table 1) with physicians from two pediatric hospitals. Boston Medical Center is a large urban safety-net hospital with pediatric capabilities where PEM physicians provide online EMS medical oversight. Boston Children’s Hospital is a free-standing children’s hospital and quaternary care center.

### Participants

Each prehospital team consisted of two paramedics to reflect local EMS practice and a PEM physician as the remote expert. Certified paramedics whose practice included scene response, and board-eligible or board-certified pediatric emergency physicians were eligible for inclusion. We excluded Emergency Medical Technicians (EMT) and Advanced EMTs as advanced life support exceeds their scope of practice. We also excluded EMS clinicians whose practice included interfacility or pediatric specialty transport, resident physicians-in-training, and non-physician providers.

### Recruitment and enrollment

Recruitment occurred from January 1, 2021 to June 30, 2022. The principal investigator (PI) gave a standardized introduction at each site. An EMS medical director, EMS training personnel, or physician collaborator distributed recruitment fliers and email invitations. Participants were protected from clinical duties and remunerated if permitted by agency rules. Paramedics received continuing education credit.

We assembled teams using convenience sampling. Prescheduled biweekly simulation sessions were offered during recruitment. Volunteers self-registered using a web-based application. Paramedics participated during paid work or overtime hours, or off-duty hours at agency discretion. Physicians participated during non-clinical hours.

### Randomization and blinding

We block-randomized teams to teleconsultation (intervention) or usual care (control) groups with 1:1 allocation. The study statistician generated the random allocation sequence. Group assignment was concealed during participant self-registration, but participants could not be fully blinded due to the intervention. In-person and video outcome assessors and statisticians were blinded to participant identity and group assignment.

### Simulation equipment

We developed a mobile “*in situ* simulation kit” to standardize simulations and data collection. The kit included audiovisual recording equipment, an infant manikin, a simulated patient monitor, and a sham medication bag. We used SimBaby™ (Laerdal Medical, Stavanger, Norway), a high-fidelity wireless and tetherless manikin with advanced clinical functions, displays of programmable vital signs, and ability to assess CPR and procedure quality.

Simulations were conducted at EMS agencies in off-duty ambulances equipped for advanced life support. Paramedics could access all emergency supplies, equipment, resources, and protocols available in usual care. We created a sham medication bag to avoid use of real medications, which paramedics restocked before each simulation to encourage familiarity. Physicians had access to printed EMS protocols and pediatric dosing references.

### Simulation procedures

Each simulation session lasted four hours. Simulation and recording equipment were staged in the ambulance. Simulations were recorded by two portable digital cameras (Logitech C920 HD Pro Webcam) from two angles using WireCast (version 14.0 software, Telestream, LLC).

The simulation team included a facilitator and an observer. The facilitator operated simulation and recording equipment, provided scripted prompts, and acted as a standardized family member during each case. The observer recorded clinician actions.

Each session began with a pre-brief to introduce the study, obtain consent, and orient participants to the simulation equipment and environment. Paramedics participated in the ambulance. Physicians participated from a separate location (e.g., home). Each team completed three pediatric cases (cardiac arrest, respiratory failure, and status epilepticus)^28^ in a standard order. Cases were concealed from participants until each simulation began. Cases represented refractory, life-threatening illness to prompt communication with medical control physicians per state EMS protocols. Paramedics reset the ambulance and sham medication bag between cases. The facilitator led a debrief summarizing learning objectives and discussing team performance after all cases and post-simulation interviews were completed.

### Intervention platform and training

**Fig. 2:**
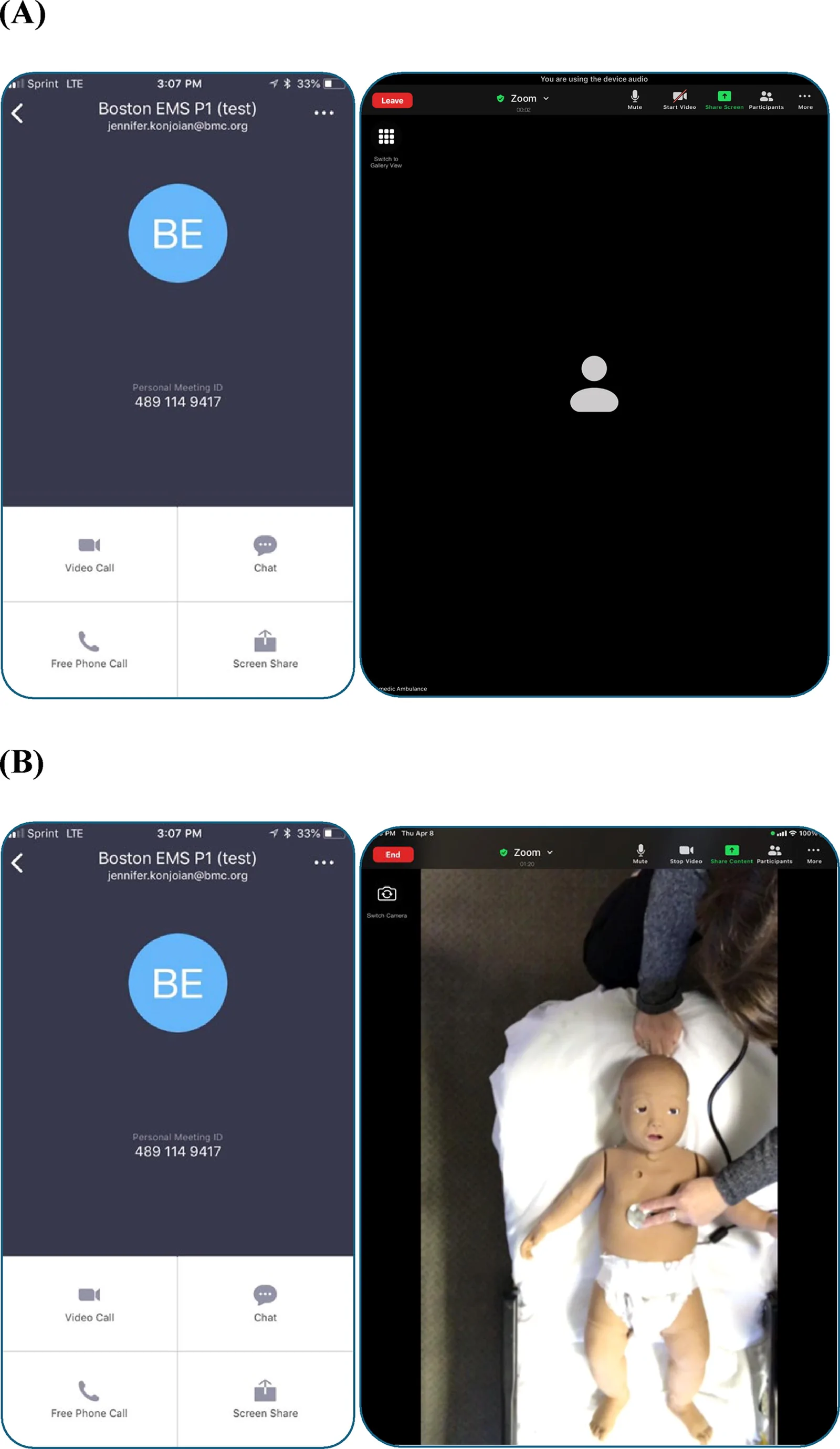
**Teleconsultation software interface**. Physician consultation was initiated using standard Zoom Pro software on mounted tablets. Appearance of the software with tablet screen for the paramedics initiating the call (left), and consulting physician during the call (right) for (A) the usual-care arm using audio only communication and (B) the intervention arm using audio and video communication.

**Fig. 3:**
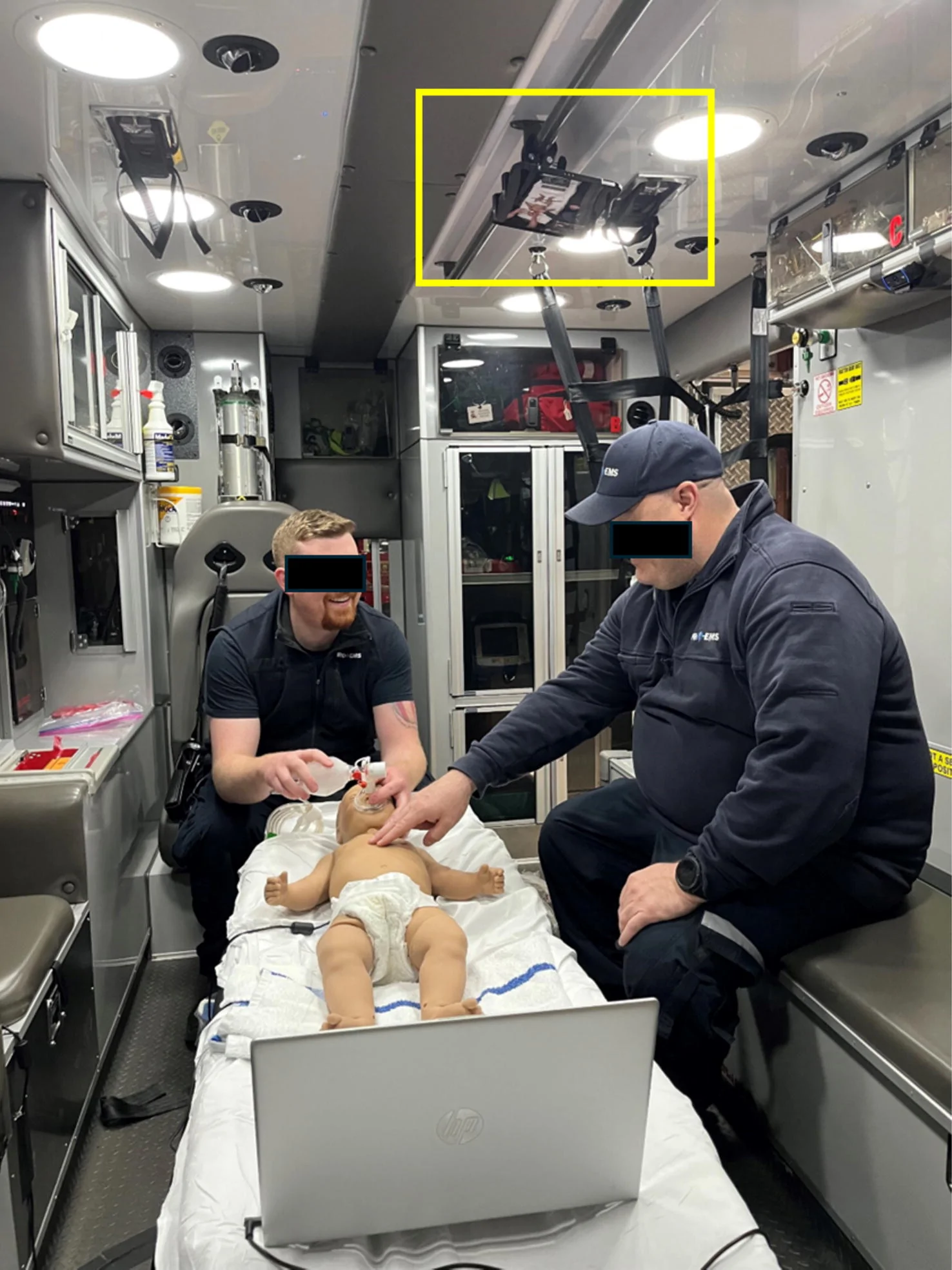
**Teleconsultation hardware setup**. Tablet is mounted with a flexible clamp on the ceiling of the ambulance to provide an aerial view of the patient (infant manikin).

Zoom Pro (Zoom Video Communications, Inc., San Jose, CA), a HIPAA-compliant video-conferencing application was preloaded on tablets (iPad, Apple, Cupertino, CA) as the teleconsultation platform because audio and video channels can be selected independently, and calls started with one touch (Fig. 2). One tablet was mounted on the ambulance roof for an aerial view of the infant manikin (Fig. 3), while the other was hand-held by the remote physician. Before pre-briefing, participants reviewed a just-in-time training graphic with instructions on platform use. During pre-briefing, they practiced making and receiving calls with feedback to demonstrate platform technical competency.

### Intervention delivery

Control paramedic dyads were instructed to provide usual care using standing orders and physician consultation via audio-call for additional orders per EMS protocol. Intervention paramedic dyads were instructed to provide usual care, but consult the physician via video-call immediately after initial patient assessment to enable visualization of the patient and actions. Paramedics selected when to initiate either call. Physicians consulted via audio- (control) or video-call (intervention).

### Data collection and measures

After simulation, each participant completed a demographic and characteristics form and the NASA Task Load Index (NASA-TLX) to measure impact on workload.^29^ Intervention arm participants also completed the Telehealth Usability Questionnaire (TUQ).^30^ The 21-item TUQ assesses telehealth usability in six domains (usefulness, ease of use, interface quality, interaction quality, reliability, and satisfaction) with 7-point Likert scales. The total usability score (TUS) is the average of all TUQ items (maximum = 7). The primary outcome, acceptability, was measured by the TUS with adequate acceptability defined *a priori* as TUS ≥ 6.^31^ The secondary outcome, feasibility, was measured by TUQ items 11 through 14 assessing interaction quality.

Quantitative data were collected and managed using REDCap electronic data capture tools hosted by Boston University (CTSI 1ULTR001430).^32^, ^33^ Two study team members conducted semi-structured qualitative interviews to assess acceptability, perceived benefits and risks, and strategies for clinical integration and mitigating adverse events. Interview audio-recordings were transcribed by a professional service.

### Analysis

We used Kraemer’s approach to pilot studies^34^ to identify a sample size range to estimate acceptability with adequate precision for future trial planning. Based on preliminary work,^28^ we used an expected mean TUS of 6 and standard deviation (SD) of 0.8 to compute 95% confidence intervals (CI) at various sample sizes. Ten teams per arm produced a TUS 95% CI of 5.4–6.6, while 20 teams per arm gave a 95% CI of 5.6–6.4.

We calculated descriptive statistics to report participant demographics, TUS, and workload scores stratified by provider type and/or study arm. We applied intention-to-treat principles for between-arm analysis. Two independent sample t-tests examined TUQ and workload score differences between physicians and paramedics. Spearman correlation analysis explored correlations between TUS and overall workload scores. All analyses were conducted using SAS version 9.4 (SAS Institute Inc., Cary, NC).

For interview data, we developed a codebook using Technology Acceptance Model constructs^35^ to capture themes associated with clinician perceptions of teleconsultation (Supplemental Table 3). Two coders reviewed and annotated three transcripts using the initial codebook. They added emergent themes not captured by the model in consensus coding meetings, refining the codebook before coding remaining transcripts. We used deductive analysis to identify themes. Qualitative data were coded and analyzed in NVivo version 12 (Lumivero, Denver, CO).

## Results

We randomized 24 paramedic-physician teams (Fig. 1). Table 1 summarizes participant demographics and prior experience with simulation training and telemedicine, stratified by provider type and study arm. Most participants had no prior experience with telemedicine for children under 12 years old in any setting, including 79% of physicians and 96% of paramedics.

**Table 1:** Participant characteristics by provider type and study arm.

**Participant characteristics**	**Physicians**			**Paramedics**		
	**Total** **(*n* = 24)**	**Usual care** **(*n* = 12)**	**Teleconsultation** **(*n* = 12)**	**Total** **(*n* = 48)**	**Usual care** **(*n* = 24)**	**Teleconsultation** **(*n* = 24)**
**Age in years**						
Median (IQR)	41.0 (36.0–47.5)	43.0 (39.5–50.0)	37.0 (34.5–43.5)	32.5 (28.5–40.0)	33.0 (28.5–38.5)	32.0 (28.5–45.5)

	***n* (%)**					
**Sex**						
Male	10 (43)	6 (50)	4 (33)	36 (75)	17 (71)	19 (79)
Female	14 (58)	6 (50)	8 (67)	12 (25)	7 (29)	5 (21)
**Race**						
Asian	3 (13)	2 (17)	1 (8)	0 (0)	0 (0)	0 (0)
Black/African American	1 (4)	0 (0)	1 (8)	1 (2)	1 (4)	0 (0)
White	17 (71)	8 (67)	9 (75)	45 (94)	22 (92)	23 (96)
Other	3 (13)	2 (17)	1 (8)	2 (4)	1 (4)	1 (4)
**Ethnicity**						
Hispanic or Latino	1 (4)	1 (8)	0 (0)	1 (2)	1 (4)	0 (0)
Non-Hispanic or Latino	23 (96)	11 (92)	12 (100)	45 (94)	22 (92)	23 (96)
Missing	0 (0)	0 (0)	0 (0)	2 (4)	1 (4)	1 (4)
**Clinical experience at current level**						
<5 years	10 (42)	3 (25)	7 (58)	20 (42)	10 (42)	10 (42)
5–9 years	5 (21)	3 (25)	2 (17)	14 (29)	8 (33)	6 (25)
10–19 years	6 (25)	4 (33)	2 (17)	8 (17)	5 (21)	3 (13)
≥20 years	3 (13)	2 (17)	1 (8)	5 (10)	1 (4)	4 (17)
Missing	0 (0)	0 (0)	0 (0)	1 (2)	0 (0)	1 (4)
**Simulation experience**^a^						
Task trainer	20 (83)	10 (83)	10 (83)	25 (52)	13 (54)	12 (50)
Manikin-based	24 (100)	12 (100)	12 (100)	40 (83)	18 (75)	22 (92)
Standardized patient	20 (83)	9 (75)	11 (92)	25 (52)	13 (54)	12 (50)
Virtual reality	13 (54)	7 (58)	6 (50)	7 (15)	4 (17)	3 (13)
None	0 (0)	0 (0)	0 (0)	6 (13)	5 (21)	1 (4)

**Any telemedicine experience**						
**For patient <12 years old**						
Never	19 (79)	8 (67)	11 (92)	46 (96)	24 (100)	22 (92)
Once	1 (4)	1 (8)	0 (0)	1 (2)	0 (0)	1 (4)
2–9 times	3 (13)	2 (17)	1 (8)	1 (2)	0 (0)	1 (4)
≥10 times	1 (4)	1 (8)	0 (0)	0 (0)	0 (0)	0 (0)
**For patient ≥12 years old**						
Never	18 (75)	8 (67)	10 (83)	40 (83)	21 (88)	19 (79)
Once	2 (8)	1 (8)	1 (8)	1 (2)	0 (0)	1 (4)
2–9 times	4 (17)	3 (25)	1 (8)	1 (2)	0 (0)	1 (4)
≥10 times	0 (0)	0 (0)	0 (0)	6 (13)	3 (13)	3 (13)

**Emergency or prehospital telemedicine experience**						
**For patient <12 years old**						
Never	21 (88)	9 (75)	12 (100)	48 (100)	24 (100)	24 (100)
Once	1 (4)	1 (8)	0 (0)	0 (0)	0 (0)	0 (0)
2–9 times	2 (8)	2 (17)	0 (0)	0 (0)	0 (0)	0 (0)
≥10 times	0 (0)	0 (0)	0 (0)	0 (0)	0 (0)	0 (0)
**For patient ≥12 years old**						
Never	21 (88)	10 (83)	11 (92)	44 (92)	21 (88)	23 (96)
Once	2 (8)	1 (8)	1 (8)	0 (0)	0 (0)	0 (0)
2–9 times	1 (4)	1 (8)	0 (0)	0 (0)	0 (0)	0 (0)
≥10 times	0 (0)	0 (0)	0 (0)	4 (8)	3 (13)	1 (4)
**Smart phone or tablet device experience**						
Yes	21 (88)	10 (83)	11 (92)	42 (88)	21 (88)	21 (88)
**Zoom video-conferencing platform experience**						
Yes	21 (88)	10 (83)	11 (92)	41 (85)	21 (84)	20 (87)

a Percentages do not sum to 100% because participants could select multiple options.

Table 2 summarizes the median ratings for each TUQ item and TUS for the intervention arm, stratified by provider type. Mean TUS was 5.6 (95% CI 5.2, 6.0) for physicians and 5.8 (95% CI 5.5, 6.0) for paramedics. The interaction quality item ratings, which reflected feasibility, were consistently above 6 for both provider types—the mean IQS was 5.2 (95% CI 4.6, 6.0) for physicians and 5.9 (95% CI 5.4, 6.3) for paramedics. Low reliability item ratings reflected the lack of error messaging in the platform to help users resolve problems.

**Table 2:** Telehealth Usability Questionnaire scores (median, IQR) for intervention arm participants by provider type.

**Domain**	**No.**	**Questionnaire Item**	**Physicians** **(*n* = 12)**	Paramedics* (*n* = 23)
Usefulness	1	Telemedicine improves my access to medical control physician consultation	7.0 (7.0–7.0)	7.0 (6.0–7.0)
	2	Telemedicine saves me time when communicating with a medical control physician	6.0 (4.5–7.0)	6.0 (5.0–7.0)
	3	Telemedicine provides for my needs as a prehospital provider	6.5 (6.0–7.0)	6.0 (5.0–7.0)

Ease of use & learnability	4	It was simple to use this system	7.0 (7.0–7.0)	7.0 (6.0–7.0)
	5	It was easy to learn to use the system	7.0 (7.0–7.0)	7.0 (6.0–7.0)
	6	I believe I could become productive quickly using this system	7.0 (7.0–7.0)	7.0 (6.0–7.0)

Interface quality	7	The way I interact with the system is pleasant	7.0 (6.0–7.0)	7.0 (6.0–7.0)
	8	I like using the system	6.0 (6.0–7.0)	6.0 (5.0–7.0)
	9	The system is simple and easy to understand	7.0 (6.0–7.0)	7.0 (6.0–7.0)
	10	This system is able to do everything I would want it to be able to do	6.0 (5.0–7.0)	7.0 (5.0–7.0)

Interaction quality	11	I could talk to the clinician easily using the telemedicine system	6.5 (5.5–7.0)	7.0 (6.0–7.0)
	12	I could hear the clinician easily using the telemedicine system	6.0 (4.5–6.5)	6.0 (5.0–7.0)
	13	I felt I was able to express myself effectively	6.5 (6.0–7.0)	7.0 (5.0–7.0)
	14	Using the telemedicine system, I can see the patient/clinician as well as if we met in person	4.0 (3.0–5.5)	6.0 (4.0–7.0)

Reliability	15	I think the consultations provided over the telemedicine system are the same as radio-based (CMED) consultations	2.0 (2.0–3.0)	2.0 (1.0–6.0)
	16	Whenever I made a mistake using the system, I could recover easily and quickly	5.0 (0.0–5.5)	5.0 (3.0–7.0)
	17	The system gave error messages that clearly told me how to fix problems	0.0 (0.0–0.0)	1.0 (0.0–5.0)

Satisfaction & future use	18	I feel comfortable communicating with the clinician using the telemedicine system	6.5 (6.0–7.0)	7.0 (6.0–7.0)
	19	Telemedicine is an acceptable way to give online medical direction for pediatric emergency care	7.0 (7.0–7.0)	7.0 (6.0–7.0)
	20	I would use this telemedicine platform again	7.0 (7.0–7.0)	7.0 (5.0–7.0)
	21	Overall, I am satisfied with this telemedicine platform	7.0 (6.0–7.0)	7.0 (6.0–7.0)

Total Usability Score (maximum score = 7)			**5.6 (5.3**–**6.1)**	**6.0 (5.2**–**6.3)**

* One paramedic did not complete the Telehealth Usability Questionnaire.

We examined adherence to and feasibility of initiating physician contact per protocol (Table 3). Usual care paramedics attempted physician contact in 47% (17/36) of simulated transports versus 94% (34/36) of intervention paramedics, with contact initiated later in the usual care arm. Ten of the 19 (53%) simulated transports without any physician contact attempt in the usual care arm involved cardiopulmonary arrest (Supplemental Table 2). When attempted, paramedics were able to connect to physicians within two tries (88% usual care vs 94% intervention; *p* = 0.59). Most (41/47, 87%) connected successfully on the first attempt within 30 s.

**Table 3:** Mean time intervals to initiate and establish physician contact, and number of attempts required to establish a successful connection during each 15-minute simulated transport, stratified by study arm.

	**Usual care** **(*n* = 36 transports)**	**Teleconsultation** **(*n* = 36 transports)**
Mean time to first call attempt, minutes (95% CI)	8.6 (7.4, 9.8)	2.6 (2.1, 3.1)
Mean time to first physician contact, minutes (95% CI)	9.2 (8.2, 10.2)	3.2 (2.5, 4.0)

**Number of attempts made to establish a connection**		
No attempts	19 (53%)	2 (6%)
1 attempt	12 (33%)	30 (83%)
2 attempts	3 (8%)	3 (8%)
≥3 attempts	2 (6%)	1 (3%)
Mean attempts to successful connection (95% CI)	1.2 (1.1, 1.3)	1.1 (1.0, 1.2)
% connection success in ≤2 attempts	15 (88%)^a^	32 (94%)^b^

a Usual care, *n* = 17.

b Teleconsultation, *n* = 34.

Table 4 summarizes physician and paramedic perceptions of workload stratified by study arm. Overall, usual care physicians reported significantly lower workload (39.4; IQR 27.7, 57.6) when compared to intervention physicians (73.7; IQR 62.3, 77.5). However, workload scores for paramedics were similar for usual care (70.7; IQR 66.6, 76.1) and intervention groups (70.0; IQR 56.5, 73.2), although intervention paramedics reported less frustration.

**Table 4:** Median participant NASA Task Load Index (NASA-TLX) scores with interquartile range (IQR) for individual domains and overall workload stratified by provider type and study arm.

**NASA-TLX domain**	**PEM physicians**			**Paramedics**		
	**Usual care** **(*n* = 10)**^b^	**Teleconsultation** **(*n* = 11)**^b^	***p*-value**^a^	**Usual care** **(*n* = 20)**^b^	**Teleconsultation** **(*n* = 22)**^b^	***p*-value**^a^
Mental demand	33 (5–60)	80 (75–90)	0.001	79 (71–83)	79 (65–85)	0.75
Physical demand	0 (0–2)	4 (0–20)	0.16	50 (30–64)	33 (12–66)	0.25
Effort	17 (5–50)	75 (74–80)	0.001	72 (67–80)	70 (50–82)	0.60
Temporal demand	19 (0–50)	70 (50–80)	0.003	75 (64–81)	65 (50–80)	0.35
Performance	68 (50–91)	75 (66–80)	>0.99	58 (50–70)	65 (50–74)	0.63
Frustration	8 (0–42)	56 (24–75)	0.02	66 (51–75)	50 (30–61)	0.03
Overall workload	39 (28–58)	74 (62–78)	0.001	71 (67–76)	70 (57–73)	0.26

a Wilcoxon rank‑sum test.

b Three teams did not complete the NASA-TLX.

Table 5 summarizes key themes with representative quotes from interviews with intervention participants. Providers reported positive experiences using teleconsultation and many favored adoption. One physician remarked “…*this was enough of a demonstration for me to think that this would be feasible….”* A paramedic said, *“I would want to use it now personally*… *we just don’t get enough critical [pediatric] calls to ever feel truly comfortable”.*

**Table 5:** Major themes and representative quotes from post-simulation interviews with intervention arm physicians and paramedics.

**Theme**	**Representative quotes from physicians**	**Representative quotes from paramedics**
***Attitudes and future intention to use teleconsultation***		
Provider attitudes were positive and favored future adoption and use.	“…this was enough of a demonstration for me to think that this would be feasible and something [like this] would be doable.” “I think overall it does have a lot of potential…”“…if the physician is working at the same time, I don’t think they can give their full attention to both the clinical space and this mobile platform.”	“…it seems like it has more benefit than it does harm…”“…I would take this right now today…”

***Ease of use of intervention technology***		
Video platform was simple and easy to use	“…it was really easy to use, super-easy to connect…”“…answering the call just connecting and suddenly being part of the team was the best feature.”	“It’s a lot easier than [using] the phone or radio…”“…one button to open it up and go right where you need to be. I thought was helpful.”
Familiarity with videoconferencing technology facilitates use and learnability	“… Zoom is a platform most of us, if not all of us are used to using by now…”	“…zoom, I feel like everybody had done a million times…so you don’t have to really think about [it].”
Video quality was adequate to visualize patient and care delivery	“…it gives you a great view of the patient… and what [the paramedics] are using…”“…from the view I had… I would be able to have a good assessment of what their respiratory status was.”	“Even though you don’t need to see [the physician], [because] you can see them, you want to see them…”“If the screen was bigger it would be more natural”
Audio quality may interfere with provider communication	“…when [the paramedics] were speaking amongst themselves, it was hard for me to hear…”	“We can hear [the physician] a lot better because he’s speaking into a microphone”“External speakers would definitely help with us being able to hear them.”

***Usefulness***		
Video platform improves access to pediatric expertise in real-time	“…providing an extra set of eyes and expertise can be helpful…”	“…highly valuable having some expert consultation right there and they can talk and see [in] real time.”“…it would increase the confidence in feeling that you’re not completely alone.”
Video platform facilitates non-verbal communication	“…[makes] it easier to call in and … show what’s going on with the patient as opposed to trying to explain…”	“They could physically see it themselves would I think make things quicker, more efficient.”
Improves physician integration into care team	“… having the video actually makes you feel a little bit more like you’re a part of the team than you would with just voice.”“…video definitely gives me more data and allows me to [be] more efficient…”	“…just like another set of eyes on the scene.”“…a higher provider whose main focus is just focus on running [the code]…”

***Potential barriers***		
Reliability of hardware, software, and broadband connectivity were a major concern	“…if one out of every 20 calls you’re having trouble connecting…that could be disastrous.” “I would worry the most about dropped calls or audio…”	“… an iPad really isn’t that sturdy…”“…I know definitely more rural places that can absolutely be dead zones for wireless connections…”
Audio/video quality in real-world settings may be limiting	“…there was no movement of the ambulance, there was no bouncing…”	“…be interesting to see how it will go in a moving truck because that’s loud”“…Road noise, parent is freaking out, like these trucks aren’t soundproof…”
Physician role in care team needs definition	“I just wasn’t quite sure about the culture of what my role was”“I felt more like I was running a resuscitation than really being a med-control physician which is hard to know how much I should be”	
Physicians require greater understandingof EMS capabilities	“I think we can learn what paramedics are capable of doing but its working with each individual that has a lot of variation”“…someone who is more versed with their kind of capability would be a little more effective.”	“…one of the frustrations I had was… the physician not knowing our protocols and scope of practice.”“I think it would save time if they [physicians] were more familiar with our protocols”
Acceptability by experienced paramedics may be limiting		“…just getting everybody on the same page, it’s gonna be the tough part.”“Change is scary. That’s one thing that will drive people away.”

These positive attitudes were partially driven by perceptions that video was useful for improving communication and enhancing physician involvement. One physician noted *“*… *having the video actually makes you feel a little bit more like you’re a part of the team than you would with just voice”.* Another clarified this sentiment, saying “…*did you give medications, are you bag-mask ventilating, have you started compressions, have you done a pulse check*… *you can get all that information visually without having to continually ask for it.”.* This indicates video enhanced physician involvement and efficiency through non-verbal communication. Paramedics echoed this, saying: *“*…*[it was] highly valuable having some expert consultation right there and they can talk and see [in] real time.”*

Providers were unaccustomed to the new team dynamic created by early physician presence. One physician stated, *“I felt more like I was running a resuscitation than really being a med-control physician, which is hard to know how much I should be”.* Another remarked, *“…someone who is more versed with their kind of capability would be a little more effective”,* highlighting that physician experience with EMS protocols and care may impact consultation effectiveness. Paramedics also noted this potential deficit, as one said *“… one of the frustrations I had was… the physician not knowing our protocols and scope of practice.”*

The video platform was easy to use, even during high-stress simulations. “…*One button to open it up and go right where you need to be*”, said one paramedic commenting on how easily the interface facilitated teleconsultation. Additionally, providers indicated that video quality was good and facilitated care. One physician remarked that the video feed “… *gives you a great view of the patient*… *and what [the paramedics] are using*…*”,* indicating the current interface was adequate for physicians to participate in care and visually assess the patient.

Audio quality was variable when environmental noise was high, which may be magnified in real-world conditions. One physician commented that, “*when [the paramedics] were speaking amongst themselves, it was hard for me to hear…*”. Paramedics did not have to decipher multiple voices, but one said it will, *“… be interesting to see how it will go in a moving truck because that’s loud.*” Another suggested “*External speakers would definitely help with us being able to hear them…*” Providers expressed uncertainty about the reliability of the technology and broadband connection. One paramedic stated ‘*“*…*I know definitely more rural places that can absolutely be dead zones for wireless connections*…*”.* As a physician noted, “*… if one out of every 20 calls you’re having trouble connecting … that could be disastrous.*”

## Discussion

This simulation study demonstrates that real-time teleconsultation by pediatric emergency physicians to support prehospital care of critically ill children is acceptable to paramedics and physicians. We successfully integrated off-the-shelf video conferencing technology into EMS workflows with a 92% connection rate overall. Paramedics reported less frustration but otherwise no workload change. However, physicians experienced increased workload, which should inform future system design. Usability scores for both provider types met the predefined acceptance threshold within 95% confidence intervals. Providers praised the system’s ease of use and valued physician integration and enhanced team communication. Still, concerns about audio reliability, broadband infrastructure, and unclear role definitions must be resolved before clinical implementation.

Improving prehospital care for children is a national priority. Pediatric teleconsultation could benefit patients, EMS clinicians and agencies, and health systems by providing real-time expert support to help EMS clinicians recognize deterioration, reduce medication and equipment errors, guide timely escalation, and improve adherence to resuscitation guidelines.^25^, ^36^, ^37^ Although outcome benefits remain to be demonstrated, just-in-time guidance could complement EMS clinician judgement and support a workforce with limited exposure to critically ill children.^38^, ^39^ Regional networks could extend pediatric expertise to low-volume EMS agencies, provided they have reliable connectivity, available consultants, appropriate training, and sustainable operating costs. Program value should therefore be evaluated alongside effects on pediatric readiness, patient safety, and quality improvement.

Prehospital teleconsultation has benefited time-sensitive adult emergencies^37^, ^40^ but remains unevaluated for children. We previously found paramedics are interested in teleconsultation for children citing provider discomfort and growing familiarity with telehealth.^20^ Our findings suggest access to pediatric teleconsultants could offload paramedic stress by shifting the cognitive burden to physicians. Given the potential physician impact, sustainable implementation will require protected coverage, clear response-time expectations, standardized escalation protocols, integrated documentation, compensation, and training in video-based consultation. Monitoring physician workload and response times and adjusting staffing or workflows accordingly could help avoid missed or delayed consultations.

Research describing when and how often EMS clinicians use physician oversight is limited. Most studies examine specific scenarios rather than system-wide rates.^41^, ^42^ Our observations support evidence that paramedics consult physicians selectively based on system design^13^ and perceptions of the interaction.^43^, ^44^ This study suggests teleconsultants experienced in pediatric emergency care and prehospital medicine may build trust and encourage EMS adoption.

Our results align with the Technology Acceptance Model, which links simplicity and usefulness to adoption.^35^, ^45^ Providers found the teleconsultation system easy to use with minimal training, noting its simple interface and one-touch call capability. We chose off-the-shelf software over a custom platform to foster broad adoption, especially in smaller EMS systems where benefits for children may be greatest.

Participants identified benefits beyond patient visualization. Video enabled non-verbal communication, let physicians observe paramedic actions, and reduced reliance on voice-only descriptions. This is consistent with telemedicine research highlighting benefits of visual cues for assessment.^46^, ^47^ Visualizing the scene also fostered team cohesion—physicians felt more integrated and paramedics valued expert guidance in high stress moments.

Both groups, however, were frustrated by unfamiliar team dynamics. Physicians were uncertain about their role and varied between “coach” and “leader”, possibly due to variable EMS education and exposure.^48^ Paramedics felt limited physician knowledge of EMS protocols reduced consultation effectiveness. These findings mirror literature showing role ambiguity in technology-mediated care can undermine performance.^49^ Establishing clear guidelines for team member responsibility will be essential to preserve paramedic autonomy while maximizing expert input.

Regional systems with centralized command structures are one way to leverage pediatric specialists. Program governance must address credentialing, workflow, physician engagement, incentives, and the added value of video over voice consultation. Regional programs should clearly define clinical authority for treatment and destination decisions; criteria for escalation or transport without consultation; responsibility when advice is delayed, disputed, misunderstood, or unavailable; and how physicians are credentialed across jurisdictions. They should also establish requirements for documentation, consent, privacy, and cybersecurity.

A cost-utility model for in-ambulance stroke telemedicine found that cost-effectiveness depends on clinically meaningful gains.^50^ Thus, patient and health system savings may not accrue to organizations funding EMS teleconsultation. Implementation costs include equipment, connectivity, personnel, and governance, some of which could be shared with existing EMS telehealth programs or 911 modernization efforts. Pediatric teleconsultation does not require wholesale 911 system redesign. A focused program could operate alongside existing voice and dispatch processes with simple devices, reliable networks, a defined medical control pathway, integrated documentation, and voice or radio backup.

Although simulations showed high connection success, providers worried about real-word reliability. Paramedics reported service interruptions with broadband devices, particularly outside city centers, which could delay care or cause harm.^51^, ^52^ To mitigate this, EMS systems should maintain fallback communications via telephone or radio. Audio quality was another concern, though ambient noise of the ambulance and medical devices did not disrupt communication in simulations. Future work should assess the impact of noise and connectivity in moving ambulances with sirens and larger teams. Other potential risks include delays from limited physician availability, miscommunication, incomplete assessment, overreliance on remote advice, and unclear clinical authority or liability. Programs should also address privacy, cybersecurity, workload, and risk that teleconsultation could displace local medical direction, pediatric readiness, and workforce training efforts.

### Limitations

This study has important limitations. Simulation cannot fully replicate real-world conditions, like broadband disruptions, excess noise, limited lighting, and vehicle motion, which may influence provider perceptions. Incorporating technical and environmental contextual factors into future simulations and field validation could improve generalizability. This study was designed to assess acceptability and feasibility, not efficacy, and was not powered for clinical outcomes; analyses of performance and medical errors will be reported separately. Voluntary recruitment may have introduced selection bias with telehealth-friendly participants. Participants were recruited from urban centers in a Massachusetts tertiary referral network, so attitudes may not generalize to those in other regions or rural settings.

### Conclusions

This proof-of-concept study shows that teleconsultation during EMS transports of critically ill children is acceptable to providers and feasible in simulation. Paramedics and physicians valued pediatric expertise and perceived enhanced teamwork and communication. The teleconsultation system was usable with adequate video and audio quality. Concerns remain about real-world reliability, including connectivity and audio interference, but may be feasible to implement with minor modifications. Clinical implementation will require: (1) testing under real-world conditions with contingency plans for technological failures; (2) clear protocols defining provider roles and decision-making authority to reduce cognitive burden and optimize team function; and (3) training to familiarize physicians with paramedic capabilities and protocols. Fully powered efficacy trials are needed to determine impact on patient safety and clinical outcomes.

## CRediT authorship contribution statement

**Tehnaz P. Boyle:** Writing – original draft, Supervision, Project administration, Investigation, Funding acquisition, Conceptualization. **Derrick Chu:** Writing – original draft, Investigation, Formal analysis. **Ijeoma M. Okafor:** Writing – original draft, Visualization, Formal analysis, Data curation. **Lauren Cronin:** Writing – review & editing, Investigation. **Caitlin Farrell:** Writing – review & editing, Validation, Investigation. **Stephanie Stapleton:** Writing – review & editing, Validation, Investigation. **Ron Medzon:** Writing – review & editing, Validation, Investigation. **Barbara M. Walsh:** Writing – review & editing, Validation, Investigation. **Howard Cabral:** Writing – review & editing, Supervision, Methodology, Formal analysis. **Erik Wang:** Writing – review & editing, Project administration. **Mari-Lynn Drainoni:** Writing – review & editing, Supervision, Methodology. **Vinay Nadkarni:** Writing – review & editing, Supervision, Methodology, Conceptualization. **Carlos A. Camargo:** Writing – review & editing, Supervision, Conceptualization. **James A. Feldman:** Writing – review & editing, Supervision, Conceptualization.

## Funding

This research was supported by the National Heart, Lung, and Blood Institute (Grant No. K23HL145126) and the National Center for Advancing Translational Sciences, National Institutes of Health, through BU-CTSI Grant Number 1UL1TR001430.

## Declaration of competing interest

The authors declare that they have no known competing financial interests or personal relationships that could have appeared to influence the work reported in this paper.

## Appendix A Supplementary material

Supplementary Data 1 This includes EMS agency characteristics, connection success by study group for each simulation case, and qualitative study materials.
