# Supplementary material for: Feasibility and acceptability of pediatric expert teleconsultation during critical ambulance transports: a pilot randomized controlled simulation trial
Source: Resusc Plus. 2026 Aug 14;31:101448. doi: 10.1016/j.resplu.2026.101448 (PMC13544342; doi:10.1016/j.resplu.2026.101448)
Supplement: Supplementary file 1 [file mmc1.docx]

**Supplemental Data**

**Supplemental Table 1.** EMS agency characteristics

| **Agency** | **Type** | **Total 911 transports/year*** | **Pediatric 911 transports/year* (%)** | **Total paramedics** |
| --- | --- | --- | --- | --- |
| Boston EMS | Municipal (City) | 87,259 | 3,979 (4.6%) | 52 |
| South Shore Hospital EMS | Private (non-profit) | 6,940 | 403 (5.8%) | 64 |
| Bedford Fire Department | Municipal (Fire) | 1,315 | 55 (4.2%) | 11 |
| Billerica Ambulance Service | Municipal (City) | 4,192 | 307 (7.3%) | 20 |
| Burlington Fire Department | Municipal (Fire) | 2,350 | 77 (3.3%) | 29 |
| *Values represent averages for 2021 and 2022. | | | | |

**Supplemental Table 2:** Connection success for audio and video calls by study group for each simulation case.

| **Case** |  | **Usual Care**  **(n=12)** | **Teleconsultation**  **(n=12)** |
| --- | --- | --- | --- |
| CPA | Mean time to 1^st^ call attempt (minutes) (SD) | 7.7 (5.2) | 3.5 (2.2) |
|  | Mean time to first physician contact (minutes) (SD) | 10.3 (2.0) | 4.8 (3.4) |
|  | Number of attempts to establish a connection |  |  |
|  | No attempts | 10 | 1 |
|  | 1 attempt | 1 | 8 |
|  | 2 attempts | 1 | 3 |
|  | ≥3 attempts | 0 | 0 |
|  | Mean attempts to successful connection (SD) | 1.5 (0.7) | 1.3 (0.5) |
|  | % connection success in ≤2 attempts | 2/2 (100%) | 10/11 (91%) |
|  |  |  |  |
| Asthma | Mean time to 1^st^ call attempt (minutes) (SD) | 7.7 (3.9) | 2.5 (1.3) |
|  | Mean time to first physician contact (minutes) (SD) | 8.5 (3.7) | 2.8 (1.4) |
|  | Number of attempts to establish a connection |  |  |
|  | No attempts | 5 | 0 |
|  | 1 attempt | 6 | 12 |
|  | 2 attempts | 0 | 0 |
|  | ≥3 attempts | 1 | 0 |
|  | Mean attempts to successful connection (SD) | 1 (0.0) | 1 (0.0) |
|  | % connection success in ≤2 attempts | 6/7 (86%) | 12/12 (100%) |
|  |  |  |  |
| Seizure | Mean time to 1^st^ call attempt (minutes) (SD) | 9.4 (3.3) | 1.8 (0.8) |
|  | Mean time to first physician contact (minutes) (SD) | 9.5 (3.0) | 2.1 (0.9) |
|  | Number of attempts to establish a connection |  |  |
|  | No attempts | 4 | 1 |
|  | 1 attempt | 5 | 10 |
|  | 2 attempts | 2 | 0 |
|  | ≥3 attempts | 1 | 1 |
|  | Mean attempts to successful connection (SD) | 1.3 (0.5) | 1 (0.0) |
|  | % connection success in ≤2 attempts | 7/8 (88%) | 10/11 (91%) |
|  |  |  |  |

**Supplemental Table 3.** Codebook Definitions

| **Usefulness** | **Healthcare provider perception that expert teleconsultation could enhance or diminish care delivery in pre-hospital emergencies** |
| --- | --- |
| Access to providers | Provider perception that technology use positively or negatively impacts access to a pediatric expert |
| Efficiency | Provider perception that technology use increases or decreases efficiency of care delivery (e.g., time, resource utilization) |
| Interaction | Provider’s perception that the technology and/or process improves or impairs interpersonal interaction or communication |
| Peer-to-peer | For peer-to-peer interaction or communication |
| Provider-to-patient | For Provider-to-patient interaction or communication |
| Quality improvement & assurance | Provider perception that technology use could positively or negatively impact the process of performance review of care delivery |
| Training & education | Provider perception that technology use could enhance or diminish provider training opportunities |
| **Ease of use** | **Provider’s perception that the technology is easy or difficult to use** |
| Simplicity | Provider’s perception that the technology platform and/or process is simple or complex |
| Learnability | Provider’s perception that the technology platform and/or process is easy or difficult to learn |
| Interface quality | Provider’s perception of the quality of the technology platform interface |
| AV quality | Provider’s perception of the quality of audio and/or video in the platform |
| Reliability | Provider’s perception that the technology is reliable or unreliable (i.e., it works as intended when you need it) |
| **Internal context** | **Internal factors such as values, characteristics, or experience that impact user attitudes** |
| Provider | Provider’s own values, characteristics, or experience |
| Organization | Provider’s hospital or care facility values, characteristics, or experience |
| Access to technology | Provider’s access to sufficient devices |
| **External context** | **Factors external to the organization (e.g., policies, resources, infrastructure)** |
| Policies, protocols, and guidelines | State or municipal policies, protocols, or guidelines related to prehospital care delivery |
| Resources | Provider’s access to internet or telecommunications networks for adequate connectivity |
| **User attitude** | **Provider’s positive or negative feeling towards the technology** |
| **Behavioral intention to use** | **Provider’s intent/willingness to use or not use the technology** |
| **Recommendations for future implementation** | **Provider’s recommendations for future implementation of technology** |
| Barriers | Provider’s perceived potential barriers to future implementation |
| Facilitators | Provider’s perceived potential facilitators of future implementation |

**Supplement: Interview Guide**

**Introductory script:** “Thank you for participating in this pilot study to test the feasibility and acceptability of using pediatric telemedicine for prehospital emergency care for children. Today, I want to gather your opinion based on your experience with a prototype telemedicine system during the simulations. As a reminder, this interview will be audio-recorded for research purposes only. We will not record any personal identifying information.”

**Semi-structured Interview Questions** (based on Technology Assessment Model Framework)**:**

1) Role identification
   a. During the pilot simulation study, were you a **paramedic** OR a **physician** **teleconsultant**?
2) User beliefs about system **usefulness**
   a. What is your **overall impression** of this prototype pediatric telemedicine system for EMS?
   b. How do you feel that this system would **support or not support** **EMS providers** to care for sick and injured children? What would be **most useful?** What would be **less useful**?
   c. What would make this system **worth using**?

3) Features supporting or hindering **ease of use**
   a. In your opinion, which elements of the prototype system **worked well**?
      i. Which **technologic features** made the system easy to use?

(*These are features of the platform interface—e.g., icon buttons, user interface)*

1. Were the icon buttons intuitive or self-explanatory regarding their function?
2. Which technologic features do you think are essential to enable use of a telemedicine platform while caring for a sick child in the prehospital setting?
ii. Which **operational features** made the system easy to use?

(*These are the processes or procedures used to operate the system—e.g., turn it on; make/receive a call; end a call*)

1. Would it be important to have a mobile platform? Why or why not?
2. What would be potential barriers to real world use if you had a mobile platform? What would be potential facilitators?
b. In your opinion, which elements of the prototype system were **challenging or could pose a barrier to patient care**?
   i. Which **technologic features** did you find challenging?
   ii. Which **operational features** could be challenging?
   iii. Which **team communication features** did you find worked well? Which were challenging?
   iv. Was your ability to communicate effectively impacted by the **lack of prior knowledge or relationship** **with the other provider**(s)? If so, how?
c. Were there any features that you would want to make the platform easier to use that were **missing**?
d. What supports or other technology would you **need to** **be productive** with this system?

4) User **intention to use** the system
   a. Would you use this (or a similar) system to **provide clinical care**?
      i. What would make you **want** to use this system again?
      ii. What would make you **NOT want** to use this system again?
   b. Based on your experience, what kind of barriers do you anticipate may **prevent adoption or use** of this or a similar pediatric telemedicine system for EMS?
   c. What would **make you want to use** a pediatric telemedicine system for EMS? (i.e. what are the most important SYSTEM attributes that would make you want to use it in a high stress environment)
5) Do you have any **additional feedback** for us?
